# Research on logistics carbon emissions under the coupling and coordination scenario of logistics industry and financial industry

**DOI:** 10.1371/journal.pone.0261556

**Published:** 2021-12-28

**Authors:** Lu Wang

**Affiliations:** School of Economics and Management, Inner Mongolia Agricultural University, Inner Mongolia Autonomous Region, Hohhot, China; Universiti Malaysia Sabah, MALAYSIA

## Abstract

In order to measure the impact of the degree of coupling and coordination between logistics and financial industries on the carbon emissions of the logistics industry, a comprehensive evaluation index system for the logistics-financial industry from 2007 to 2017 was constructed, which was calculated using the multi-objective linear weighting method; and a combination of logistics and financial industry was constructed. The panel smooth conversion model of coordination degree and carbon emission of logistics industry. The empirical results show that the coupling coordination degree of logistics and financial industry in China’s 30 provinces is mostly in primary and medium coordination, and only a few provinces are on the verge of imbalance; the coupling coordination degree in the eastern region is overall moderate coordination, and the overall coordination degree in the central and western regions It is the primary coordination; with the growth of GDP per capita, the degree of coupling and coordination between the logistics and financial industries has promoted the increase of carbon emissions in various regions, but the promotion effect is an inverted U-shaped trend that first increases and then decreases.

## Introduction

With the continuous development of the Internet and e-commerce, the demand for the logistics industry has also increased, and the integration of the financial industry and the logistics industry has continued to deepen, gradually forming a symbiotic relationship that is mutually conditional and mutually motivating. In March 2019, the National Development and Reform Commission and other ministries and commissions issued the "Opinions on Promoting the High-quality Development of Logistics and Promoting the Formation of a Strong Domestic Market." Financial institutions or large logistics enterprise groups initiate logistics industry development investment funds". Driven by the e-commerce and financial industries, the scale of the logistics industry has continued to expand. According to data released by the China Federation of Logistics and Purchasing, my country’s total social logistics reached 298 trillion yuan in 2019, a year-on-year increase of 5.9%. However, with the continuous expansion of the scale of the logistics industry, the carbon emissions generated in the process of logistics operations are gradually increasing, and a huge "external negative effect" has been produced. With the improvement of living standards, people are becoming more and more aware of the importance of green logistics. Energy conservation and emission reduction in the logistics industry have become the key to achieving high-quality economic development. In this process, the financial industry provides an important supporting role for the green transformation and development of the logistics industry. How to achieve a win-win situation for the economic and ecological benefits of the logistics industry through the integration of the logistics industry and the financial industry has also become the focus and difficulty that needs to be solved urgently problem.

At home and abroad, the integrated development of the logistics industry and the financial industry is mainly focused on the coordinated development relationship between the logistics industry and the financial industry. Zhou and Yang (2012) [1] discussed the inevitable trend and practical significance of the coordinated development of the logistics industry and the financial industry. The research of Silvestro and Lustrato (2014) [2] shows that the orderly collaboration and information sharing in the supply chain integration system cannot be separated from the assistance of banks to benefit buyers and suppliers. Ding Yongqi (2014) [3] empirically analyzed the dynamic relationship between the logistics industry and the development of the financial industry by constructing a vector autoregressive model, studied the ways and means of the financial industry to promote the development of the logistics industry, and put forward suggestions for the development of the logistics industry. Zhang Jianjun and Zhao Qilan (2018) [4] based on the characteristics of the development of my country’s logistics industry and financial industry, used a dynamic coupling coordination degree model to analyze the coordinated development relationship between the logistics industry and the financial industry. The research results show that the coordination of China’s logistics industry and the financial industry Sex has gone through a process of change from uncoordinated to barely coordinated to coordinated. Chu Xuejian and Qian Sainan (2019) [5] constructed a comprehensive evaluation index system for the logistics industry and the financial industry, and calculated the degree of coupling and coordination between my country’s logistics industry and the financial industry from 2003 to 2017. The level of coupling and coordination is gradually rising. With the insufficient expansion of the logistics scale, the concept of "low-carbon logistics" has gradually attracted the attention of academia and the government. Low-carbon logistics originates from a low-carbon economy in order to reduce the impact of the logistics industry’s carbon emissions on the environment. As one of the industries with high energy consumption, the logistics industry needs to consider low-carbon development, that is, reducing transportation, warehousing, distribution, information and other processes carbon emission. Li Xiaohui (2019) [6] used the entropy weight coupling model to measure the degree of coupling and coordination between China’s financial industry and logistics industry from 2003 to 2017. The results show that China’s financial industry and logistics industry are both developing well. Zhou Qiqing (2021) [7] used the double random effect model to study finance, logistics, and economic development, and measured the coupling degree of finance and logistics. Through the research, it was found that finance and logistics promote economic development and the coordinated development of finance and logistics. importance. Geng Liyan (2020) [8] constructed a model to measure the degree of coupling between the logistics industry and the financial industry from 2003 to 2017, and obtained the factors that have the greatest impact on the logistics industry and the financial industry, and the process of their coordinated development.

Xue Yang et al. (2019) [9] used a three-stage DEA model to construct a logistics efficiency evaluation system under carbon emission constraints. The study found that the logistics efficiency value is related to the environment and random interference factors, and evaluated the development level of the logistics industry in 10 key provinces and cities. did an evaluation. Hu Xiaofei et al. (2019) [10] used the carbon footprint method to measure and use the gray correlation model to analyze related influencing factors based on the carbon emission data of the logistics industry in 2006–2016. The total amount of emissions fluctuates and rises as a whole. Ren Guoqiang (2021) [11] used the Gini coefficient method to analyze the carbon emissions of China’s logistics industry from the perspective of carbon sources, and concluded that oil is the main reason for the differences in carbon emissions in various regions. Based on the financial crisis and the development history of the logistics supply chain, Liu Weihua [12] used the likert scale questionnaire collection method to analyze the important segmentation strategy of encouraging foreign trade logistics companies to develop supply chain services.

Through the above analysis, it can be seen that the related research on the coupling and synergy between the logistics industry and the financial industry and the carbon emissions of the logistics industry has achieved richer research results. However, few documents incorporate the coordinated development of the logistics industry and the financial industry and the carbon emissions of the logistics industry into a unified research framework, and analyze the impact of the integrated development of the logistics industry and the financial industry on the carbon emissions of the logistics industry. Is it linear or non-linear? Taking this as a starting point, this paper separately measured the coupling and coordination degree of the logistics industry and the financial industry and the carbon emissions of the logistics industry in 30 provincial administrative regions (except Tibet) in China from 2007 to 2017, and incorporated the two into a unified model. The panel smooth conversion model is used to test the relationship between the two, to provide a certain reference for the green development policy of the logistics industry in various regions.

## Materials and methods

### Index system construction

The sequence parameters in the movement of the logistics-financial complex system have two characteristics: ① Determine the macroscopic phenomenon of the system and indicate the level of the system’s order; ② The system generates and determines the final structure and sequence of the system. Select the parameters that meet the characteristics and substitute them into the model to solve, and the selected order parameters should have the characteristics of representativeness, macroscopic, global, usability, and a combination of dynamic and static. Drawing on the existing literature, following the principles of scientificity, data availability, and maneuverability, this paper constructs a comprehensive rating index system for the logistics subsystem and the financial subsystem, as shown in Table 1. The logistics subsystem and the financial subsystem are set as level I indicators, including three aspects: operational indicators, scale indicators and development indicators. Among them, there are 10 level II indicators under the logistics subsystem and 11 level II indicators under the financial subsystem. Level indicators. The data comes from the "China Statistical Yearbook" and the official website of the National Bureau of Statistics. And the selected order parameters should have the characteristics of representativeness, macroscopicity, globalization, usability, and a combination of dynamic and static. Drawing on the existing literature, following the principles of scientificity, data availability, and maneuverability, this paper constructs a comprehensive rating index system for the logistics subsystem and the financial subsystem, as shown in Table 1. The logistics subsystem and the financial subsystem are set as level I indicators, including three aspects: operational indicators, scale indicators and development indicators. Among them, there are 10 level II indicators under the logistics subsystem and 11 level II indicators under the financial subsystem. Level indicators.

**Table 1:** Comprehensive evaluation index system of logistics industry subsystem and financial industry subsystem.

System Level	I Index Level	I Index Weight Level	II Index Index	Name Level	II Index Weight
Logistics Subsystem	Operational indicators	0.30167	L1	Transport mileage	0.10115
			L2	Number of postal outlets	0.09991
			L3	Number of employees	0.10061
	Scale index	0.3911	L4	Freight volume	0.09774
			L5	Cargo turnover	0.09340
			L6	Value Added	0.09914
			L7	Investment in fixed assets	0.10082
	Developmental indicators	0.30721	L8	Value added as a proportion of GDP	0.10250
			L9	The added value accounts for the proportion of the tertiary industry	0.10206
			L10	Employed persons account for the proportion of employed persons in urban units in the province	0.10265
Financial subsystem	Operational indicators	0.45371	F1	Year-end deposit balance	0.08918
			F2	Year-end loan balance	0.08873
			F3	Original insurance premium income	0.09177
			F4	Original insurance premium expenditure	0.09139
			F5	Number of employed persons in urban units of the financial industry	0.09265
	Scale index	0.26863	F6	Financial correlation rate (deposit + loan balance/GDP)	0.09084
			F7	Insurance depth = premium income/GDP	0.09066
			F8	Value Added	0.08713
	Developmental indicators	0.27766	F9	Value added as a proportion of GDP	0.09004
			F10	The added value accounts for the proportion of the tertiary industry	0.09415
			F11	Employed persons account for the proportion of employed persons in urban units in the province	0.09347

## Empirical model construction and variable measurement

### Model construction

#### Panel smooth conversion model

González et al. (2005) [13] extended the panel threshold model proposed by Hansen (1999) [14], so that the change of regression coefficients between different zoning systems is no longer a jump, but a smooth gradual process. It is more in line with economic reality, and a panel smooth transition (PSTR) model is proposed. The most basic two-mechanism panel smooth conversion model is as follows:

$$y_{it}=\mu_{i}+\beta_{0}^{'}x_{it}+\beta_{1}^{'}x_{it}g(q_{it};\gamma ,c)+\varepsilon_{i}$$ (1)

Among them, i = 1, 2,…, N, t = 1, 2,…, T, is the conversion function of the transfer variable, and it changes continuously between [0,1]; the regression coefficient vector is the conversion The continuous function of the variable is the conversion speed of the conversion between different zoning systems; c is the position parameter where the conversion occurs; borrowing from existing research, the conversion function selects the logical function (He Xiaoli and Pan Haoran, 2013 [15]) form:

$$g(q_{ij};\gamma ,c)={\left(1+\exp(-\gamma \overset{m}{\underset{j=1}{\Pi }}(q_{ij}-c_{j}))\right)}^{-1}$$ (2)

The general form of the smooth conversion model of multi-system panel is:

$$y_{it}=\mu_{i}+\beta_{0}^{'}x_{it}+\sum_{j=1}^{r}\beta_{j}^{'}x_{it}g_{j}(q_{it}^{j};\gamma ,c)+\varepsilon_{it}$$ (3)

The meaning of the parameters is the same as formula (1). In this article, represents the carbon emissions of the logistics industry in each region, represents the degree of coupling and coordination between the explanatory variable logistics and the financial industry and the vector of control variables, and the conversion variable is the per capita GDP level of each region.

#### Carbon emission estimation model for logistics industry

The carbon emissions of the logistics industry are represented by the CO2 emissions of the transportation, storage and postal industries. Since there is currently no release of CO2 emission data for each province and industry, this article draws on the calculation method of Chen Shiyi (2011) [16], and uses the primary energy consumption and the carbon emission coefficient in the IPCC to estimate. The specific calculation formula is:

$$CO_{2}=\sum_{i=1}^{7}CO_{2i}=\sum_{i=1}^{7}E_{i}*NCV_{i}*CEF_{i}*COF_{i}*\frac{44}{12}$$ (4)

Among them, represents the consumption of the i-th primary energy (i = 1, 2,…7); represents the average low calorific value of the i-th primary energy; represents the carbon emission reference coefficient of the i-th primary energy; represents The carbon oxidation factor of the i-th one-time energy, 44 is the molecular weight of CO2, and 12 is the molecular weight of carbon. The data comes from the "China Energy Statistical Yearbook" and IPCC reports.

#### Description of other variables

Conversion variable: Expressed by the per capita GDP of each province and deflated with 2000 as the base period. The remaining control variables include regional government fiscal expenditure, expressed as the proportion of total fiscal expenditure in total output value (TE); the tax revenue of each regional government, expressed as the proportion of total tax revenue in total output value (TT); each region Industrial structure: expressed by the proportion of the secondary industry in the total output value; technical level of each region: expressed by the proportion of the regional technology market turnover in GDP. The data comes from the "China Statistical Yearbook".

### Measurement and calculation of coordination degree of logistics and finance coupling

#### Coupling degree of logistics and finance

This paper incorporates the logistics industry and the financial industry into a unified analysis system, constructs a comprehensive evaluation index system for the logistics subsystem and the financial subsystem, and uses the multi-objective linear weighting method to calculate the comprehensive evaluation index of the logistics industry and the financial industry. The specific calculations are as follows:

(1) Standardized processing

First, the range standardization method is used to standardize the raw index data of the logistics subsystem and the financial subsystem:

Positive indicators:

$$y_{j}=(x_{j}-x_{j\min})/(x_{j\max}-x_{j\min})$$ (5)

Reverse indicators:

$$y_{j}=(x_{j\max}-x_{j})/(x_{j\max}-x_{j\min})$$ (6)

Among them, *x*_*j*_ represents the value of index j, *x*_*j*max_ and *x*_*j*min_ represents the maximum and minimum values of the index respectively.

(2) Determination of index weight

The entropy weight method is used to determine the weight of each indicator. First, calculate the indicator weight:

$$p_{ij}=x_{ij}/\sum_{i=1}^{m}x_{ij}$$ (7)

Among them, m represents the number of provincial regions. Secondly, calculate the entropy of index j:

$$e_{j}=-k\sum_{i=1}^{m}p_{ij}\times \ln\;p_{ij}$$ (8)

In formula (7), k = 1/lnm, therefore, 0 ≤ e ≤ 1. Again, calculate the utility value of each indicator:

$$d_{j}=1-e_{j}$$ (9)

Among them, the larger the value, the greater the corresponding weight. Finally, calculate the weight of the indicator:

$$w_{j}=d_{j}/\sum_{j=1}^{m}d_{j}$$ (10)

(3) Calculate the comprehensive index of the logistics industry and the financial industry

This paper uses the multi-objective linear weighting function method to calculate the comprehensive evaluation index of the logistics and financial industries:

$$Logi_{i}=\sum_{j=1}^{n}w_{j}x_{xj}$$ (11)

$$F{\mathrm{ina}}_{i}=\sum_{j=1}^{n}w_{j}x_{ij}$$ (12)

Among them, and are between 0–1, the larger the value, the better the development status, and vice versa.

(4) Coupling and coordination degree of logistics and financial industry

Apply the coupling relationship to the interaction between the logistics industry and the financial industry, namely:

$$C={\left\{f(U)g(E)/{\left[(f(U)+g(E)/2)\right]}^{2}\right\}}^{1/2}$$ (13)

Further construct the coupling and coordination degree model of logistics and financial industry to analyze the coupling and coordination relationship between logistics and financial industry:

T=αf(U)+βg(E) (14)

$$D=\sqrt{CT}$$ (15)

Among them, Eq (13) represents the logistics industry subsystem, represents the financial industry subsystem, C represents the degree of coupling between logistics and financial industry, T represents the comprehensive coordination index of the logistics industry and financial industry, and D represents the degree of coupling and coordination between logistics and financial industry. α and β represent the contribution shares of the logistics industry and the financial industry, respectively.

#### Coupling and coordination classification

To more directly reflect the degree of coupling and coordination between the logistics industry and the financial industry, this paper draws on the classification of coupling coordination types by Zeng Fanqing and Ye Dezhu (2017) [17], and based on the coupling coordination degree and the comprehensive index of the logistics subsystem and the financial subsystem. The degree of coupling and coordination between the logistics and financial industries is divided into 5 categories, and the 5 categories are further divided into 10 sub-types. The results are shown in Table 2.

**Table 2:** Types of coupling between global value chain embedding and ecological environment.

Degree of coordination	Coordination D	Coupling coordination classification	*g*(*E*) and *f*(*U*) Relations with size and basic types
Advanced coordination	0.90−1.00	High-quality coordinated development	(1) *g*(*E*)>*f*(*U*) and *g*(*E*)−*f*(*U*)>0.1, Financial industry lagging; (2) 0≤|*g*(*E*)−*f*(*U*)|≤0.1, Streaming Finance Synchronous; (3) *g*(*E*)<*f*(*U*) and *g*(*E*)−*f*(*U*)<0.1, Logistics industry lagging
	0.80−0.89	Well coordinated development	
Intermediate coordination	0.70−0.79	Intermediate coordinated development	
Basic coordination	0.60−0.69	Primary coordinated development	
	0.50−0.59	Barely coordinated development	
Overtype	0.40−0.49	On the verge of dysregulation	
	0.30−0.39	Mild dysregulation	
Dysregulation	0.20−0.29	Moderate Disorder Recession	
	0.10−0.19	Severe dysregulation and recession	
	0.00−0.09	Extremely maladjusted recession class	

## Results and discussion

### Analysis of coordination degree of logistics finance coupling

In order to more accurately analyze the degree of coupling and coordination between the logistics subsystem and the financial subsystem, this paper draws on the processing method of Wang Shaojian et al. (2015) [18] and examines the three different shares of the logistics subsystem and the financial subsystem (α = 1/3)., β = 2/3; α = 1/2, β = 1/2; α = 2/3, β = 1/3), based on three situations, the degree of coupling and coordination of logistics finance was measured, 2007–2017 The average calculation results of each province (municipalities directly under the Central Government) in the year are shown in Table 3. It can be seen from Table 3 that the coupling coordination degree measured under the three different contribution shares is basically the same, indicating that the difference in the contribution share has little effect on the logistics financial coupling degree model. In this paper, α = 1/2, β = 1 /2, that is, the case where the contribution shares of the logistics subsystem and the financial subsystem are respectively 0.5 are analyzed as an example. It can be seen from Table 3 that the coupling coordination degree of the logistics industry and the financial industry in the 30 provinces (municipalities directly under the Central Government) is mostly in the barely coordinated and primary coordination stage. There are still more provinces at the end of the imbalance stage, and Hainan and Qinghai provinces It is in a stage of mild dysregulation. From the perspective of the eastern, central, and western regions, the overall degree of coupling and coordination in the eastern region is in the primary coordinated development category, the central region is in the barely coordinated development category, and the western region is in the dysfunctional and recessive category as a whole. It can be seen that the logistics and financial industries in the eastern region The degree of coupling coordination is higher than that of the central and western regions. From the perspective of sub-types, most of the central and western regions have lagging or synchronized development of the financial industry, while Beijing, Shanghai, and Zhejiang in the eastern region have lagged behind the logistics industry, indicating that the logistics industry in most provincial-level regions is relatively more developed than the financial industry. Better, and the financial industry in Beijing, Shanghai and Zhejiang Province has developed more rapidly.

**Table 3:** The degree of coordination between the logistics industry and the financial industry in each province (municipalities directly under the Central Government) under different coupling types.

province	Type one: *α* = 1/3,*β* = 2/3	Type two: *α* = 1/2,*β* = 1/2	Type three: *α* = 2/3,*β* = 1/3	Type
Beijing	0.6449	0.6981	0.7476	Intermediate coordination-logistics lagging
Tianjin City	0.4842	0.4905	0.4964	On the verge of dysfunction-synchronous development
Hebei Province	0.6808	0.6472	0.6116	Primary coordination-financial lag
Shanxi Province	0.6192	0.6167	0.6141	Primary coordination-synchronous development
Inner Mongolia Autonomous Region	0.5556	0.5125	0.4654	Barely coordinated-financial lagging
Liaoning Province	0.6296	0.6114	0.5925	Primary coordination-financial lag
Jilin Province	0.4442	0.4288	0.4126	On the verge of dysfunction-synchronous development
Heilongjiang Province	0.5194	0.4979	0.4752	Barely coordinated-financial lagging
Shanghai	0.7027	0.7340	0.7640	Intermediate coordination-logistics lagging
Jiangsu Province	0.7308	0.7359	0.7410	Intermediate coordination-synchronous development
Zhejiang Province	0.6538	0.6749	0.6953	Primary coordination-logistics lag
Anhui Province	0.5461	0.5368	0.5273	Barely coordinated-synchronous development
Fujian Province	0.5542	0.5364	0.5180	Barely coordinated-financial lagging
Jiangxi Province	0.4823	0.4574	0.4309	On the verge of dysfunction-financial lag
Shandong Province	0.7154	0.6954	0.6747	Intermediate coordination-financial lagging
Henan Province	0.6279	0.6054	0.5821	Primary coordination-financial lag
Hubei Province	0.5926	0.5669	0.5400	Barely coordinated-financial lagging
Hunan Province	0.5703	0.5428	0.5138	Barely coordinated-financial lagging
Guangdong Province	0.8213	0.8188	0.8162	Good coordination-synchronous development
Guangxi Zhuang Autonomous Region	0.5079	0.4841	0.4591	On the verge of dysfunction-financial lag
Hainan	0.3724	0.3599	0.3467	Mild Disorder-Synchronous Development
Chongqing	0.5549	0.5515	0.5480	Barely coordinated-synchronous development
Sichuan Province	0.6602	0.6503	0.6404	Primary coordination-synchronous development
Guizhou Province	0.5155	0.4803	0.4424	Barely coordinated-financial lagging
Yunnan Province	0.4731	0.4695	0.4657	On the verge of dysfunction-synchronous development
Shaanxi Province	0.5355	0.5216	0.5073	Barely coordinated-synchronous development
Gansu province	0.4432	0.4280	0.4120	On the verge of dysfunction-synchronous development
Qinghai Province	0.3931	0.3894	0.3854	Mild Disorder-Synchronous Development
Ningxia Hui Autonomous Region	0.4629	0.4635	0.4640	On the verge of dysfunction-synchronous development
Xinjiang Uygur Autonomous Region	0.4769	0.4635	0.4496	On the verge of dysfunction-synchronous development
East area	0.6355	0.6366	0.6367	Primary coordinated development
Central Region	0.5509	0.5295	0.5068	Barely coordinated development
Western Region	0.5023	0.4902	0.4774	On the verge of dysregulation

### Analysis of the impact of logistics finance coupling coordination degree on the logistics industry’s carbon emissions

#### Model checking

First, perform linearity test and residual non-linearity test on the model. If the test result is to reject the null hypothesis (the original hypothesis is *H*_0_:*r* = 0), then the model is considered to be non-linear, and the residual non-linearity test is further performed. Table 4 shows the linearity test and nonlinearity test results of the model. It can be seen from the second column in Table 4 that in the linearity test part, the three statistics (LM, LMF, LRT) all reject the null hypothesis at a significance level of 1%, indicating that the model is non-linear, and further residuals are needed. Non-linearity test. The final test results are shown in the last column in Table 4. The three statistics in the model all accept the null hypothesis of r = 2, indicating that the optimal number of transfer functions of the model is 2.

**Table 4:** Linearity test and residual nonlinearity test results.

Statistics	Linearity test: H0:r = 0; H1: r = 1	Residual nonlinearity test: H0: r = 1; H1: r = 2	Residual nonlinearity test: H0: r = 2; H1: r = 3
LM	55.605***	25.327***	5.926
	(0.000)	(0.000)	(0.313)
LMF	11.956***	4.738***	1.024
	(0.000)	(0.000)	(0.404)
LRT	60.893***	26.352***	5.980
	(0.000)	(0.000)	(0.308)

Note: The original hypothesis H0:r = 0, that is, there is no nonlinear effect. The LM statistics obey the asymptotic *χ*^2^ distribution, the LMF obeys the *F*(*mk*,*TN*−*N*−*m*(*k*+1)) distribution, r is the number of transfer functions, the P value in parentheses, *** is the significance level of 1%, ** is the significance level of 5%, and * is the significance level of 10% Significance level.

After determining the number of conversion functions, it is also necessary to determine the number of conversion position parameters in each conversion function, that is, the value of m. Normally, m = 1 or m = 2 is selected. This article draws on Lu Yanfang et al. (2019) According to the method of AIC and BIC, the optimal m value is selected according to the minimum criterion of AIC and BIC. The test results are shown in Table 5 below. It can be seen from Table 5 that when the number of conversion positions of each conversion function is 1, the values of AIC and BIC are less than the value when the number of conversion positions is 2. Therefore, the number of the optimal conversion position of each conversion function is 1. As shown in the last column of Table 5, the number of optimal conversion functions of the model in this paper is 2, and the number of optimal conversion positions of each conversion function is 1.

**Table 5:** Determination of the number of optimal location parameters.

index	m = 1	m = 2	in conclusion
AIC	-5.633	-5.624	r = 2; m = 1
BIC	-5.415	-5.383	

#### Analysis of regression results

Table 6 shows the regression results of the panel smoothing transfer function model. It can be seen from Table 6 that the impact of the degree of coupling and coordination between the logistics and financial industries on the carbon emissions of the logistics industry has a dual-threshold feature regarding the level of per capita GDP. When the per capita GDP level is less than the first threshold point (28,105 yuan), the coupling and coordination degree of logistics and financial industries have an impact coefficient of 0.2835 on the carbon emissions of the logistics industry, which is significantly positive at a significance level of 1%; When the GDP level reaches the first threshold point, the positive promotion effect of the coupling and coordination degree of logistics and financial industry on the carbon emissions of the logistics industry in various regions increases to 1.19045 (0.2835+1.8139*0.5), and this trend continues until it is completely exceeded. Threshold, the coupling and coordination degree of logistics and financial industries have a stable impact on the carbon emissions of regional logistics industries at 2.0974 (0.2835+1.8139). Subsequently, when the per capita GDP level reached the second threshold point (33,381 million yuan), the coupling and coordination degree of logistics and financial industries weakened the role of promoting carbon emissions in the logistics industry in various regions to 1.6166 (0.2835+1.8139–0.9616*0.5). Finally, after completely crossing the second threshold, the coefficient of influence of technological innovation on the upgrading of the industrial structure stabilized at 1.1358 (0.2835+1.8139–0.9616). From the regression results, it can be seen that the coupling and coordination degree of logistics and financial industries mainly stabilizes the three zoning systems, and the impact coefficients are 0.2835, 2.0974 and 1.1358 respectively. It shows that with the growth of per capita GDP, the degree of coupling and coordination between logistics and financial industries has always had a positive impact on the carbon emissions of the logistics industry in various regions, and the positive effect has an inverted U-shaped trend that first increases and then decreases. It shows that the coupling and coordination of logistics and finance has increased the carbon emissions of the logistics industry in various regions. The main reason is that with the continuous development of the financial industry, the rise of e-commerce, various shopping platforms have gradually emerged, and the scale of the logistics industry has expanded. Due to the scale effect, the carbon emissions of the logistics industry have gradually increased. However, with the gradual increase in the level of per capita GDP, it is believed that the requirements for the quality of life and the environment have also continued to increase, and the integration of the financial industry and the logistics industry has gradually tended to green development, and the increase in carbon emissions of the logistics industry has been suppressed.

**Table 6:** The regression results of the degree of coordination between the logistics industry and the financial industry on the logistics industry’s carbon emissions.

variable	index	Influence coefficient	variable	index	Influence coefficient
Coupling degree of logistics finance	$\beta_{0}^{'}$	0.2835*** (4.6681)	Industrial structure	$\beta_{0}^{'}$	-0.0851 (-1.3959)
	$\beta_{1}^{'}$	1.8139*** (6.5130)		$\beta_{1}^{'}$	-0.1590** (-2.1407)
	$\beta_{2}^{'}$	-0.9616** (-3.2214)		$\beta_{2}^{'}$	-0.0927 (-1.1963)
Fiscal expenditure	$\beta_{0}^{'}$	-0.3022*** (-5.9348)	technique level	$\beta_{0}^{'}$	1.6342** (2.2171)
	$\beta_{1}^{'}$	0.6080* (1.6597)		$\beta_{1}^{'}$	-6.5917*** (-3.1941)
	$\beta_{2}^{'}$	0.3364 (0.8004)		$\beta_{2}^{'}$	3.9331** (2.4000)
Revenue variable	$\beta_{0}^{'}$	0.6665** (2.4876)		*c*_1_	2.8105
				*c*_2_	3.2808
Coupling degree of logistics finance	$\beta_{1}^{'}$	-8.2714*** (-4.1087)		*γ*_1_	3.3846
				*γ*_2_	9.8071
Fiscal expenditure	$\beta_{2}^{'}$	8.4973*** (4.4062)		RSS	0.988
				AIC	-5.633
				BIC	-5.415

Note: t-values are in parentheses, and *, **, *** indicate the significance levels of 10%, 5%, and 1%, respectively.

From the point of view of control variables, the influence coefficient of fiscal expenditure on the carbon emissions of the logistics industry is -0.3022, 0.3058 and 0.6422 in the three zoning systems, indicating that as the per capita GDP level increases, the increase in fiscal expenditure has promoted the growth of carbon emissions in the logistics industry. The influence coefficient of fiscal revenue on the carbon emissions of the logistics industry is 0.6665, -7.6049, and 0.8924 in the three zoning systems, indicating that as the per capita GDP level increases, the impact of fiscal revenue growth on the logistics industry’s carbon emissions is first positive, and then Turn to negative inhibition, and then turn to positive. The impact coefficient of industrial structure on the carbon emissions of the logistics industry in the three zoning systems are -0.0851, -0.2441 and -0.3368, respectively, indicating that with the increase in the per capita GDP level, the industrial structure (the proportion of the secondary industry) will affect the carbon emissions of the logistics industry. The influence is always negative, and the negative inhibitory effect gradually increases, which is in line with expectations. The influence coefficients of technological development level on carbon emissions in the three zoning systems are 1.6342, -4.9575, and -1.0244, respectively, indicating that with the increase in per capita GDP, the impact of technological level on carbon emissions in the logistics industry is first positive, and then becomes negative, and the negative impact effect shows a downward trend.

## Conclusions

With the continuous development of the financial industry and the Internet, a complex interactive coupling relationship has formed between the logistics industry and the financial industry. At the same time, as the scale of the logistics industry continues to expand, low-carbon logistics and green logistics have become important issues that the current logistics industry needs to solve. In order to analyze the impact of the integrated development of the logistics industry and the financial industry on the carbon emissions of the logistics industry, this paper constructs a comprehensive evaluation index system to measure the development level of the logistics industry subsystem and the financial industry subsystem and calculates the 30 provinces using the linear multi-objective weighting method. Comprehensive evaluation index for the coordinated and coordinated development of the logistics industry and the financial industry in high-level regions. At the same time, by constructing a panel smooth conversion model, the nonlinear effect of the coupling and coordination degree of the logistics industry and the financial industry on the logistics industry’s carbon emissions is analyzed. The research results show that: (1) The degree of coupling coordination between the logistics industry and the financial industry in 30 provinces (municipalities directly under the Central Government) is mostly at the barely coordinated and primary coordination stage, and many provinces are at the end of the stage of imbalance, while Hainan and Qinghai are at a slight level. The stage of imbalance and recession; (2) From the perspective of the eastern, central, and western regions, the overall degree of coupling coordination in the eastern region is primary coordination, the central region is in the category of barely coordinated development, and the western region as a whole is on the verge of dysregulation and recession. It can be seen that the degree of coupling and coordination of the logistics and financial industry in the eastern region is higher than that in the central and western regions. (3) The degree of coupling and coordination between the logistics and financial industries has promoted the increase in carbon emissions in various regions, but it has an inverted U-shaped trend in which the size of the role first increases and then decreases. As the per capita GDP level increases, the financial industry and the logistics industry The integration of the logistics industry is gradually moving towards a green transformation, and the increase in carbon emissions in the logistics industry has been suppressed.

Based on the above research conclusions, the policy recommendations are as follows: 1. Strengthen macro-control, strengthen government guidance, and improve the policy system related to green logistics. Establishing a sound policy system for the use of green logistics is an important guarantee for the development of green logistics. The central and local governments at all levels should actively promote and implement various green environmental protection tax preferential policies, and supervise financial institutions to support energy-saving and emission-reduction projects by formulating binding financial policies., Encourage banks to develop green credit, give tax incentives and financial subsidies to logistics companies with better energy conservation and emission reduction, and guide more funds to flow to the green logistics industry. 2. Deepen the reform of the financial system and guide the green transformation of logistics enterprises through green finance. The empirical results of this article show that the integrated development of the logistics industry and the financial industry has expanded the scale of the logistics industry, leading to an increase in carbon emissions. Therefore, how to realize green logistics through green finance is an important part of achieving high-quality economic development. First, the innovation of green logistics financial products should be enhanced, and low-carbon emission reduction in logistics should be achieved by developing financial products that are conducive to the development of green logistics; second, green car credit should be developed to provide logistics companies with sufficient capital expenditures to update clean energy transportation equipment, Speed up the green improvement of transportation equipment. Finally, establish a local green logistics bank to solve the financing problems of logistics enterprises and expand the effective supply of green finance. 3. Pay attention to the characteristics of resource endowments in the eastern, central and western regions, and formulate differentiated green logistics financial development policies. Compared with the central and western regions, the logistics and financial industries in the eastern region have developed rapidly. When formulating green logistics and green financial policies, the national and local governments should fully consider the industrial structure, development level, and human capital acquisition of each region. The government should provide more financial and technical support to the central and western regions where the integration of logistics and financial industries are relatively backward. For the eastern regions where the logistics and financial industries are well developed and have a high degree of integration, the government should make further efforts. Strengthen internal management and improve the ability to withstand financial crises. At the same time, strengthen technical exchanges and cooperation between regions, eliminate barriers to the flow of green emission reduction technologies between regions, and achieve coordinated promotion between different regions.

The coordinated development of the logistics industry and the financial industry is a necessary development trend in the future. At this stage, the level of development of the two is not satisfactory. The coordinated development of various regions is at a relatively poor stage. The important role of the two has not been played out and needs to be strengthened. Coordinated development mechanism, so that they can play the maximum effect. Promote the development of the logistics industry and the financial industry, reduce logistics carbon emissions, and reduce the differences in the development of various regions, to achieve a better coordinated development situation between the part and the whole.
